# Complex primary hips for total hip replacement surgery at a tertiary institution in Nigeria

**DOI:** 10.1051/sicotj/2018026

**Published:** 2018-06-12

**Authors:** Udo Ego Anyaehie, Gabriel Okey Eyichukwu, Cajetan Uwatoronye Nwadinigwe, Amechi Uchenna Katchy

**Affiliations:** 1 National Orthopaedic Hospital, Enugu Nigeria; 2 University of Nigeria Teaching Hospital, Enugu Nigeria

**Keywords:** Complex primary hip, Total hip replacement, Arthroplasty

## Abstract

*Introduction*: Total hip replacement (THR) surgery is still evolving in Nigeria with increasing awareness as more cases are being done. This has attraction for individuals who hitherto had no solutions for their hip pathologies. These are mostly complex primary hips which present challenging technical difficulties with increased risk of complications, thus requiring detailed planning to ensure successful operation. This work aims to present the pattern of complex primary hips presenting for THR, the challenges and complications.

*Methodology*: Data collected over a seven year period, of patients who presented for THR, were analyzed for age, sex, diagnosis, type of hip, complications, duration of surgery, blood loss and transfusions, challenges and outcome.

*Results*: Fifty-nine (43.4%) of the 136 cases of THR done were complex primary hip replacement surgeries. Avascular necrosis of femoral head amongst sickle cell disease patients (23.7%) was the commonest cause of complex primary hips in our series. Most of them had absent/tight medullary canals. This is followed by old unreduced hip dislocation and non-united hip fractures with an incidence of 10.1% each. The major peri operative complication noted was calcar split in 10 patients (16.9%)

*Discussion*: Sickle cell disease patients presented more with complex primary hips and the commonest difficulty was recreating medullary canals. Increased operation time and blood loss alongside technical difficulties should be anticipated and measures put in place to avert complications.

## Introduction

Any total hip replacement (THR) where there is an increased risk of intra operative technical difficulties and complications should be considered complex [1]. Therefore by extension, a complex primary hip is a challenging hip in which one anticipates intra operative technical difficulties and complications that should be adequately prepared for and prevented. Sathappan has described complex primary total hip arthroplasty (THA) as primary THA in patients with compromised bony or soft-tissue states, including but not limited to dysplastic hip, ankylosed hip, prior hip fracture, protrusio acetabuli, certain neuromuscular conditions, skeletal dysplasia, and previous bony procedures about the hip [2]. Some works had alluded to having a good number of complex primary hips [3] and associated difficulties [4]. In an environment like ours where late presentation to hospital is the trend [3,5] a number of these complex hips present frequently. With increasing awareness of joint replacement services in the country, patients harboring “bad” hips have started appearing in our health institution seeking arthroplasty. The aim of this study is to present the pattern of complex primary hips in our environment, perioperative challenges and complications noticed in the course of the hip replacement.

## Materials and methodology

Records of patients with complex primary hips that had arthroplasty from November 2008 to November 2015 were analyzed for age, sex, diagnosis, type of hip, challenges and complications, duration of surgery, blood loss and transfusions, and outcome. The total number of cases that had arthroplasty was noted. The surgeries were done by different surgeons and all went through the same arthroplasty protocol for the hospital. Lateral approach was used by all the surgeons. Hip prostheses from same company Depuy Johnson and Johnson were used. Regional anaesthesia was used for majority of the patients except where that failed, then general anaesthesia done. The same standard rehabilitation protocol was used except were some complications occurred, like calcar split, necessitating some modifications. Extended prophylactic antibiotic was given to all the patients. Prevention of deep vein thrombosis was by use of enoxaparin and physical methods. The clinical outcomes were analyzed using clinical scores (Harris hip score), and radiological evaluation at 6 weeks, 3 months, one year and two years. Results were analyzed using SPSS 20. Ethical approval was received.

## Results

**Figure 1:**
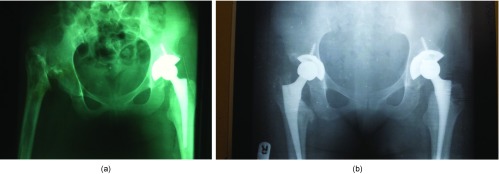
(a) Preoperative X-ray with resection arthroplasty of the right hip and THR prosthesis insitu left hip. (b) Post operative X-ray after THR of the right hip.

Fifty-nine cases of complex primary hips were operated upon. This constitutes 43.4% of the total 136 number of THR done within the period. The distribution of the complex primary hips is shown in Table 1. Figures 1a and b show preoperative and post operative X-rays respectively of a sickle cell disease patient with a right hip excision arthroplasty done in childhood.

**Table 1:** Complex primary hip cases.

Complex hip	Frequency	Percentage
Avascular necrosis of head of femur from sickle cell disease	14	23.7%
Old unreduced hip dislocation	6	10.2%
Old hip fracture non-union	6	10.2%
Excision arthroplasty hips (Girdlestone)	4	6.8%
Osteoarthritis post fracture with hard ware insitu	4	6.8%
Old non united acetabular fracture	4	6.8%
Secondary osteoarthritis post hip fracture/trauma	4	6.8%
Protrusio acetabuli	4	6.8%
Hip dysplasia	3	5.1%
Avascular necrosis of head of femur from trauma	3	5.1%
Avascular necrosis of head of femur from steroid abuse	3	5.1%
Secondary osteoarthritis post Slipped upper femoral epiphysis	2	3.4%
Avascular necrosis of head of femur from old septic arthritis	1	1.7%
Old unreduced hip dislocation with ankylosis	1	1.7%

Total	59	100%

50.8% of the cases were males and 49.1% females; giving a male: female ratio of 1:1. Age ranged from 18 to 76 with a mean of 44.6 years. The right hip was involved in 25 (42.4%) patients and left in 34 (57.6%). Duration of pain prior to presentation ranged from one year to 54 years with a mean of 9.2 years. Table 2 shows intra operative findings/challenges and solutions/maneuvers to overcome them. Most of the patients had a combination of these findings/challenges noted intra operatively. Revision cage system plus bone grafting with cemented elite plus cup was done for three cases with old acetabular fractures and one case of protrusio acetabuli.

**Table 2:** Intraoperative findings.

Intraoperative findings	Frequency	Solution offered
Severely contracted soft tissues (abductors, adductors, iliopsoas)	33	Soft tissue releases
Tight/absent medullary canal (seen more in sicklers {sickle cell disease patients})	13	Use of smallest reamer, size 6; drill bit creation of canal; mandatory use of C-arm
Pannus filled acetabulum	11	Identification of acetabulum; recreation of acetabulum
False acetabulum (seen in patients with old dislocations)	7	Identification and reaming of true acetabulum
Multiple acetabular cysts	6	Impaction autogenous bone grafting from head of femur
Acetabular protrusio	4	Impaction autogenous bone grafting from head of femur ± cage
Old non united acetabular fracture	4	Autogenous bone graft ± cage
Contained acetabular defects	2	Impaction autogenous bone grafting
Widened acetabulum	2	Jumbo shell ± impaction grafting
Uncontained acetabular defects	2	Structural bone graft with screw fixation

The duration of surgery ranged from 2 to 5 h with an average duration of surgery of 3.13 h. Blood loss ranged from 0.5 to 5 l with a mean of 1.6 l. An average of 2.5 units of blood was given to the patients, range of 1–6 units. Intra operative complications were calcar split in 10 (16.9%), femoral perforation in 2 (3.4%), acetabular perforation in 2 (3.4%). Post operative complications with intervention given are shown in Table 3. The characteristics of the hips that dislocated were: dysplastic hips (2), old non united acetabular wall fractures (2), osteoarthritis post fracture with hard ware insitu (2), and old fracture neck of femur (1).

**Table 3:** Post operative complications.

Post operative complication	Frequency/percentage	Intervention given
Dislocation	7	Revision of cup
Common peroneal nerve injury (transient) in 2 patients with marked shortening following attempt at leg length equalization	2	Positioning of knee in flexion; physical therapy; neurotropic drugs; orthotics
Pneumonia (in a sickler)	1	Pharmacothrerapy/physiotherapy
Pressure sore	1	Wound care, physiotherapy
Stem abutting on lateral cortex (in a sickler)	1	Masterly inactivity
Superficial wound infection	1	Antibiotics/wound care

The challenges experienced while operating on the patients included

- difficulty locating the medullary canals in sicklers;
- difficult reduction of proximally migrated femurs especially with old unreduced hip dislocations;
- locating the true acetabulum in patients with prior excision arthroplasty;
- difficult/ failure of hard ware removal and seeking alternative approaches where the hard ware is in the way of the prosthesis;
- avoiding acetabular floor perforation in patients with protrusio acetabuli;
- avoiding periprosthetic fractures in osteoporotic patients;
- reconstructing acetabular walls in patients with old acetabular fractures and acetabular dysplasia;
- extensive soft tissue contracture releases, bony deformities and limb length inequality corrections.

The pre op Harris hip score ranged from 8 to 76 with a mean of 39.3 and a post op Harris hip score mean of 93.75 (81–100) at 1 year. Serial radiological evaluation up until 2 years follow up for all has shown no signs of loosening. There was no mortality recorded. No infection recorded. Early outcome was satisfactory in 98.3%. Mean follow up of 4.2 years (2–9 years).

## Discussion

The prevalence of complex primary THA in our series is 43.4%. No previous study in our environment had reported on the prevalence.

Our study found majority of the complex hips in sickle cell disease patients with type 4 Ficat and Arlet Avascular Necrosis of heads of femurs with severe soft tissue contractures, limb shortening, poor bone stock and compromised medullary cavities. This is probably because sickle cell disease is quite common in our sub region and is associated with Orthopaedic pathologies of which osteonecrosis is quite common [6]. Old unreduced hip dislocation in our series presented with severe soft tissue contractures, massive shortening and challenging reduction of the head into the cup. These hips had been dislocated for between 6 months and 3 years forming a false acetabulum above the true acetabulum with pannus filled true acetabulum. The interventions are as shown in Table 2. Good results had also been reported by other workers following THR [7,8]. The patients with excision arthroplasty hips had the surgical operation done several years (1–54 years) before presentation for arthroplasty. They also had limb shortening, soft tissue contractures and pannus filled acetabulum. The hard wares encountered insitu included angle blade plates, acetabular reconstruction plates, hemiarthroplasty components. These had to be removed in the same stage surgery after which the total hip components were inserted. One patient with acetabular plate had the implant left behind as it did not interfere with prosthesis. Protrusio acetabuli posed the challenges of difficulty dislocating the hip and thin medial acetabular walls. The femoral necks were cut insitu in some cases while all had impaction bone grafting of the floor of the acetabulum. Protrusio cup was used in one patient similar to some reports [1,9]. The other complex cases as listed in Table 1 had a combination of features as represented in Table 2 which contributed to the complexities of the arthroplasty. Those with severe soft tissue contractures, massive shortening and difficulty reducing the head into the cup had 360° capsular releases as well as other soft tissue releases. None of our patients had femoral shortening in order to reduce the dislocation as reported by some workers [10]; conversion to girdlestone arthroplasty [11] was also not done on any patient. Tight or absent medullary cavity were overcome by carefully drilling into the canal. Multiple acetabular cysts and defects where carefully curetted and bone graft impacted. Unstable or non united acetabular fractures were mostly bone grafted and acetabular cage inserted with cemented elite plus cup similar to reports by these researchers [1,2,9]. Bone grafting was done using autogenous bone graft as there is none availability of allogenic bone graft (bone banks) in our centre. Location of true acetabulum in old hip dislocation and girdlestone hips was difficult but once identified, the pannus was excised, excessive bleeding which was anticipated was controlled and blood transfused as necessary. Similar intraoperative findings and solution offered have also been reported by some authors [1,2,9]. Our mean age was close to that seen in another work [12]. The mean duration of symptoms before presentation represents the usual pattern of late presentation in the study environment [3,5]. This accounts for why the hips become complex because by the time they eventually present, the pathology has worsened.

Our average duration of surgery is comparable to a study done by Clark [13]. It was high due to the technical difficulties encountered in the course of the surgery because of the pathology involved. This also may have contributed to increased blood loss in this study and thus the need for more blood transfusions. Compared to works on primary THR where mean operative time of 89 min [14] and 123 ± 28 min [15], duration in our study is higher as expected. Mean intra operative blood loss of 1600 ml is higher than 1090 ml, 984 ml and about 371 ml respectively reported in primary THR [14–16].

Out of the 10 patients with calcar split, 4 were sicklers, 3 had old hip dislocation and fractured during difficult hip reduction due to the high riding femurs. The rest were non-united hip fracture 1, girdlestone hip 1 and secondary osteoarthritis post hip fracture 1. High incidence of calcar split was mainly seen in sicklers whose poor bone quality and absent/narrow medullary cavity made reaming and stem introduction difficult. These reasons along with difficulty locating the medullary cavity led to femoral perforation in this same group of patients. Thus some works have reported that intraoperative consideration of bone stock, quality and method of component fixation may help minimize the risk of eccentric reaming, perforation or fracture of either the acetabulum or the femur, and loosening [17]. Patients with calcar split had cerclage wiring of the proximal femur and were placed on non weight bearing for 6 weeks. The patients that had femoral perforation did not need any further intervention. The canal was eventually located and stem bypassed the area of perforation. Acetabular perforation occurred in a sickler and a young man with steroid induced AVN. The perforation occurred during reaming due to extensive irregularity and weak acetabular floor respectively. Al-Mousawi reported acetabular perforation, femoral perforation and fracture similar to our report in sicklers [18]. Transfusion difficulty in this group of patient going for hip replacement surgery remains an issue as exchange blood transfusion is still considered necessary [19].

## Conclusion

The incidence of complex primary hips appears high in our sub region. It presents challenging technical difficulties with high incidence of intraoperative complications, increased operation time and blood loss. These must be anticipated and avoided as much as possible. Modalities to circumvent these problems and revision arthroplasty components must be readily available as they might be required for the primary procedures. Complex primary hips were commoner in sickle cell disease patients and the commonest surgical challenge was recreating medullary canals. We suggest routine protection of the calcar with cerclage wire intra operatively in the patients at risk of calcar split.

## Conflict of interest

The authors declare that they have no conflict of interest in relation to this article.
